# Response to Gunst and Casaer on the letter to the editor “Is the protein intake saturated at doses recommended by the feeding guidelines for critically ill patients?”

**DOI:** 10.1186/s13054-018-2189-4

**Published:** 2018-12-04

**Authors:** Arthur Raymond Hubert van Zanten, on behalf of the study group, Laurent Petit, Jan de Waele, Hans Kieft, Janneke de Wilde, Peter van Horssen, Marianne Klebach, Zandrie Hofman

**Affiliations:** Department of Intensive Care Medicine, Gelderse Vallei Hospital, Willy Brandtlaan 10, 6716 RP Ede, The Netherlands

We thank Drs Gunst and Casaer for their interest in our study showing that a very high intact-protein enteral formula (VHPF) can successfully provide protein intakes according to nutritional recommendations in overweight critically ill patients [1]. The authors speculate, by roughly estimating and referring to post hoc data from the EPaNIC-trial using parenteral nutrition, that two-thirds of the additional protein provided could be wasted in urea, potentially inducing harm. We were asked to calculate the cumulative amount of nitrogen retained [2].

Our study investigated protein and energy intake, gastrointestinal tolerance, and safety of a new VHPF (8 g/100 kcal) compared with a standard high intact-protein (5 g/100 kcal) enteral formula (SHPF). As an exploratory parameter, urinary nitrogen excretion measured on day 5 was 24 g/day in the VHPH group and 18 g/day in the SHPF group. Cumulative calculations, as suggested by Gunst, cannot be performed based on single 24-h measurements. Moreover, individual urinary nitrogen measurements could not be adequately matched with nutritional intake. Consequently, calculations based on extrapolations and assumptions will produce unreliable conclusions.

Previous studies showed that higher protein intake seems to result in improved nitrogen balance despite higher urinary urea excretion [3]. The suggested harmful effect on renal function was not demonstrated in our study.

While high protein intake is essential, the recent Protinvent study [4] suggests a slower build-up in the first ICU days should be considered. Furthermore, for most ICU patients, initial conservative energy provision has been recommended to reduce the risks of gastrointestinal intolerance and refeeding syndrome, and possibly to prevent autophagy deficiency [5].

**Fig. 1:**
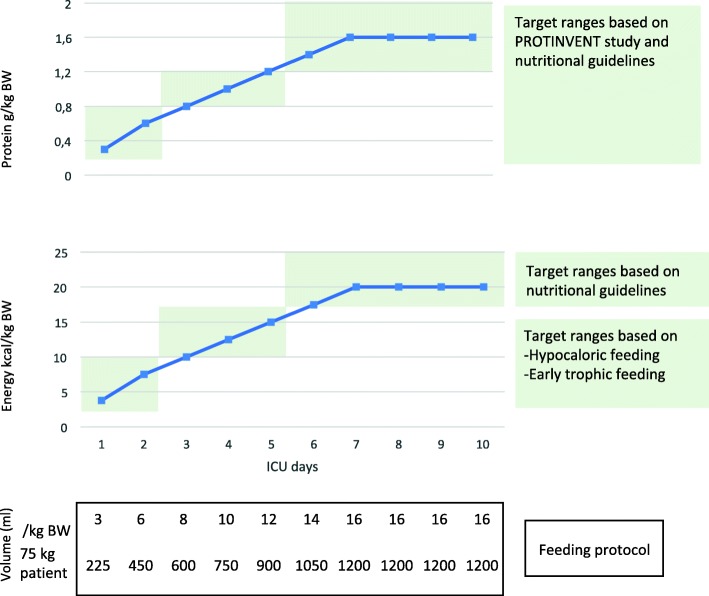
Example of simple-personalized feeding regimen (Protinvent-based protocol) with very high protein enteral nutrition (8 g protein/100 kcal, 1.25 kcal/ml) for a parallel increase in energy and protein intake in line with recommended intakes. *BW* body weight

Consequently, increasing both energy and protein in parallel may be most favorable to meet nutritional needs throughout the various phases of ICU stay. Figure 1 depicts how these nutritional targets can be achieved with a single VHPF.

In conclusion, our study showed high protein intakes according to guidelines did not result in adverse renal consequences. We suggest that a very high protein product is required to achieve an optimal energy–protein balance using a personalized build-up feeding strategy, without excessive or harmful high protein intakes or overfeeding risk.

Further investigation of protein dosing and timing on nitrogen retention and clinical outcomes is warranted. Definitive conclusions about high protein intake during critical illness can be made only based on confirmatory research, including well-designed tracer studies.

As an example, a feeding regimen with a gradual increase in calories towards target during the first week and an increase in protein intake over time was found to be associated with the lowest mortality in the Protinvent study [3]. Intake (days 1–2, < 0.8 g/kg/day; days 3–5, 0.8–1.2 g/kg/day; from day 5 > 1.2 g/kg/day) is depicted as milliliters per kilogram body weight per day and milliliters per day for a 75-kg patient.
